# Distribution and activity of nitrate and nitrite reductases in the microbiota of the human intestinal tract

**DOI:** 10.1111/febs.70299

**Published:** 2025-11-05

**Authors:** Natalie Hager, Mélanie Elisabeth Gindt, Marcel Hövels, Jean‐Lou Christian Michel Dorne, Uwe Deppenmeier

**Affiliations:** ^1^ Institute of Microbiology and Biotechnology University of Bonn Germany; ^2^ Methodology and Scientific Support Unit European Food Safety Authority (EFSA) Parma Italy

**Keywords:** gut microbiota, human intestinal tract, microbiome, nitrogen metabolism, nitrosamines

## Abstract

The human intestinal microbiota plays a vital role in health. One of the most protective benefits is the bacterial nitrogen metabolism of gut bacteria, which reduces nitrate (NO_3_^−^) and nitrite (NO_2_^−^) to ammonia or nitric oxide, preventing the formation of carcinogenic nitrosamines. In this study, we shed light on the gut bacterial NO_2_^−^/NO_3_^−^ degradation, its efficacy, and the effects on the steady‐state NO_2_^−^ concentration in the human colon. Highly abundant gut bacteria that represent the most prominent phyla were analyzed for their potential to reduce NO_2_^−^ or NO_3_^−^. *Escherichia coli* showed the greatest efficiency, which indicates a key role in the detoxification and prevention of nitrosamine formation. Species of the genera *Bacteroides* and *Phocaeicola* also contributed to NO_2_^−^ reduction due to their high abundance. The total activity of stool samples was about 620 μmol NO_2_^−^ h^−1^, indicating that NO_2_^−^ concentration in the human stool should be very low. We also show that bacterial NO_2_^−^ reduction is necessary to allow NO_2_^−^‐sensitive microorganisms to colonize the intestine, preventing a pathological shift in the composition of the intestinal microbiota. The results illustrate that the gut microbiota plays a central role in NO_2_^−^ detoxification, ensuring microbiota integrity and potentially preventing nitrosamine formation and gut‐associated cancers.

## Graphical abstract

Nitrate from food is first converted to nitrite by oral bacteria and then further processed in the intestine. The gut microbiota reduces nitrate and nitrite to ammonia or nitric oxide, preventing the formation of carcinogenic nitrosamines. Our analysis highlights *Escherichia coli* as a key player in this detoxification process, supported by other abundant bacteria like *Bacteroides* and *Phocaeicola*. This underscores the microbiota's vital role in nitrosamine prevention and gut health.

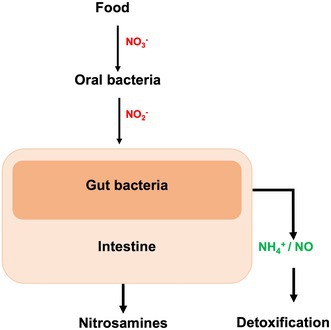

**Abbreviations**
A.: *Agathobacter*
B.: *Bacteroides*
BHI: brain–heart infusion
BLASTp: Basic Local Alignment Search Tool for proteins
DW: dry weight
E.: *Escherichia*
F.: *Faecalibacterium*
gDW: g dry weight
GIT: gastrointestinal tract
GSH: glutathione
H.: *Hominimerdicola*
IMG: Integrated Microbial Genomes and Microbiomes
K.: *Klebsiella*
L.: *Limosilactobacillus*
NA: naphthylamine
NapA: periplasmic nitrate reductase
NarG: respiratory nitrate reductase
NasA: assimilatory nitrate reductase
NasB: assimilatory nitrite reductase
NH_4_^+^
: ammonium
NirB: assimilatory nitrite reductase
NirK: copper‐containing nitrite reductase
NirS: cytochrome cd1 nitrite reductase
NO: nitric oxide
NO_2_^−^
: nitrite
NO_3_^−^
: nitrate
NOC: nitroso compounds
NrfA: respiratory nitrite reductase
OD_600_
: optical density at 600 nm
P.: *Pseudomonas*
R.: *Roseburia*
S.: *Streptococcus*
SA: sulfanilic acid
SHIME: Simulator of the Human Intestinal Microbial Ecosystem
V.: *Veillonella*
YtfE: di‐iron nitrite reductase

## Introduction

The average adult human body harbors around 10^14^ prokaryotic cells, with the majority residing in the gastrointestinal tract (GIT). This population of microorganisms constantly interacts with the gut epithelium and the immune system of the host. Thus, the gut microbiota plays a pivotal role in maintaining intestinal health, modulating the immune system, and influencing the development of both intestinal and non‐intestinal diseases. Therefore, it is of great interest to elucidate the metabolic properties of the microbiota. This is particularly relevant for nitrogen metabolism, which has already received much attention concerning the nitrogen cycle in the environment. However, the analysis of nitrogen metabolism in the GIT lags behind [1], and little is known about the corresponding reactions and enzymes of gut bacteria (except for *Escherichia* (*E.*) *coli*). As a basic element for life, nitrogen is an essential component for biomolecules, such as amino acids, proteins, and nucleic acids. Nitrogen exists in several oxidation states, ranging from +V as in the nitrate anion (NO_3_^−^) to ‐III as in the ammonium ion (NH_4_^+^). The most notable nitrogen compound conversions are: (i) denitrification (NO_3_^−^ to N_2_), whereby nitrate is successively transformed to nitrite (NO_2_^−^), nitrogen monoxide (NO), dinitrogen monoxide (N_2_O), and dinitrogen (N_2_). (ii) Nitrate/nitrite ammonification (NO_3_^−^ or NO_2_^−^ to NH_4_^+^). (iii) Nitrite reduction processes involve a novel class of nitrite reductases, referred to as di‐iron reductases [2, 3].

The catalytic intermediates of nitrate, such as nitrite and its derivatives (e.g., N_2_O_3_ and NO^+^), are important for the formation of N‐nitroso compounds (NOC) from secondary amines. Numerous investigations involving mice and rats exposed to nitrite through their diet and drinking water have consistently demonstrated increased rates of both benign and malignant tumors across various organ locations [4]. This body of research provides substantial evidence supporting the potential carcinogenicity of nitrite in the human gastrointestinal system. Leach *et al*. [5] proposed at least two distinct mechanisms for the endogenous formation of N‐nitroso compounds. The first mechanism involves a direct chemical reaction between secondary amines and nitrite, which exhibits strong pH dependence and does not occur rapidly under neutral pH conditions. The second mechanism relies on bacterial catalysis for N‐nitrosation. Published data indicate that NOC formation could be catalyzed by bacterial enzyme systems and occur significantly faster under neutral pH conditions compared to the chemical reaction [6]. This observation suggests particular relevance *in vivo*, where neutral pH, bacteria, and elevated nitrite concentrations come together. Hence, the concentration of nitrite in the GIT and the potential detoxification of nitrate/nitrite by bacterial enzymes is of crucial importance for the formation of nitrosamines. These compounds, which classify as potential carcinogens and mutagens, are important in gastrointestinal pathophysiology and can form in the stomach. In addition, nitrosamines have also been detected in human feces [7, 8].

Numerous enzymes for the nitrite and nitrate reduction are already known. Broadly, nitrate reduction is catalyzed by different classes of enzymes [9, 10]: The first class of enzymes (Nar family) comprises membrane‐bound nitrate reductases (e.g., NarG from *E. coli*). The second class contain periplasmic NO_3_^−^ reductases (e.g., NapA from *E. coli*). Both enzyme types are part of anaerobic respiratory systems. The third family includes assimilatory nitrate reductase in the cytoplasm (e.g., NasA from *Klebsiella* (*K*.) *oxytoca*). Nitrite‐reducing enzymes can also be categorized into different classes [11]. The initial class consists of enzymes that convert NO_2_^−^ to NO as part of the denitrification pathway (NirK, NirS). The second class comprises enzymes that directly convert NO_2_^−^ to NH_4_^+^. Examples are assimilatory nitrite reductases (e.g., NasB from *K*. *oxytoca* or NirB from *E. coli*) and dissimilatory nitrite reductases (e.g., NrfA from *E. coli*, NirK from *Pseudomonas* (*P*.) *chlororaphis*, and NirS from *Pseudomonas* (*P*.) *stutzeri*). In addition, di‐iron nitrite reductases exist, which are involved in detoxification processes.

In this study, we analyzed the nitrogen metabolism of the gut microbiota, focusing on the enzymatic pathways involved in nitrate and nitrite reduction. Understanding these metabolic processes is essential, as they play a crucial role in maintaining gut health, modulating microbial activity, and influencing various physiological and pathological conditions in the gastrointestinal tract.

## Results

### Bioinformatic analysis of the distribution of nitrate/nitrite reductases in gut bacteria

A bioinformatic analysis of 113 genomes of species belonging to the most important genera of the human gut was performed (Table S1). The results indicated that genes encoding NO_3_^−^ or NO_2_^−^ reductases are not widespread among these microorganisms, suggesting that NH_4_^+^ or N‐containing organic compounds are used for N‐supply. Mostly, organisms belonging to the phylum Proteobacteria (*Aeromonas*, *Aggregatibacter*, *Aliarcobacter*, *Bilophila*, *Cedecea*, *Citrobacter*, *Desulfovibrio*, *Edwardsiella*, *Escherichia*, *Haemophilus*, *Hafnia*, *Klebsiella*, *Morganella*, *Parasutterella*, *Plesiomonas*, *Proteus*, *Providencia*, *Raoultella*, *Simonsiella*, and *Yokenella*) possess genes encoding different types of NO_3_^−^ or NO_2_^−^ reductases which are involved in respiratory electron transport systems (NarG, NrfA, NapA) [1] (Table S1). However, species belonging to these genera are usually present in very small numbers within the gut microbiota, with the exception of *E. coli*.

A total of 20 members from the most abundant genera present in the human intestine were selected (Table 1) and analyzed in this study. Nitrate reductases were only found in *E. coli* and *Staphylococcus* (*S*.) *warneri* as well as in the small intestinal bacteria *Limosilactobacillus* (*L*.) *fermentum* and *Veillonella* (*V*.) *atypica*. Nitrite reductases were identified in *E. coli* and the *Bacteroides* species. Only the novel YtfE nitrite reductase showed a broader distribution, encompassing the *Bacteroides* and *Phocaeicola* species, as well as *E. coli*. Evidence of denitrifying pathways was not found in our strain collection since the genomes lacked the genes encoding the key enzymes NirK or NirS for this pathway. Such enzymes were only identified in the gut bacteria *Neisseria macacae* strain ATCC 33926 and *Simonsiella muelleri* strain ATCC 29453 (Table S1), both of which are very rare in the large intestine (abundance <0.01%). Hence, it is evident that the reduction of nitrate to N_2_ and the denitrification process are of minor importance for the nitrogen metabolism of gut bacteria. In comparison, Ravcheev and Thiele [1] examined the distribution of respiratory reductases bioinformatically in more than 200 genomes of human gut bacteria. They found that only 28 genomes, primarily from Enterobacteriaceae, encoded all enzymes for nitrate reduction to ammonia. The complete denitrification pathway, reducing nitrate to molecular nitrogen, was identified only in a *Ralstonia* strain. While genes encoding nitrate reductases were present in *Lactobacillus* and *Veillonella* species, these genomes lacked the corresponding nitrite reductase genes. In contrast, many genomes from the order Bacteroidales encoded only the periplasmic respiratory nitrite reductase Nrf. Hence, these findings are consistent with the results presented here.

**Table 1:** Distribution of nitrate and nitrite reductases in gut bacteria used in this study^a^.

	Strain	IMG genome	NasA	NarG	NapA	NrfA	NirB	NirS	NirK	YtfE
Species (small intestine)										
*Limosilactobacillus fermentum*	ATCC 14931	643 886 061		644 291 054						
*Limosilactobacillus reuteri*	ATCC 23272	640 427 118								
*Streptococcus hyointestinalis*	NCTC 12224	2 990 232 454								
*Streptococcus thermophilus*	ATCC 19258	8 115 039 888								
*Streptococcus vestibularis*	ATCC 49124	649 990 016								
*Veillonella atypica*	ATCC 17744	2 529 292 891		2 530 405 622						
*Veillonella magna*	DSM 19857	2 523 231 015		2 523 275 114						
*Veillonella rattii*	ATCC 17746	2 868 207 868		2 868 208 161						
Species (large intestine)										
*Agathobacter rectalis*	A1‐86	650 377 936								
*Akkermansia muciniphila*	BAA‐835	642 555 104								
*Bacteroides cellulosilyticus*	CRE21	643 886 111				644 167 678				644 168 023
*Bacteroides xylanisolvens*	XB1A	650 377 911				650 527 210				650 523 269
*Bifidobacterium longum*	ATCC 15697	643 348 516								
*Escherichia coli* K‐12	MC4100	2 645 728 047		2 648 092 394	2 645 728 047	2 648 090 905	2 648 090 010			2 648 091 051
*Faecalibacterium prausnitzii*	A2‐165	645 951 831								
*Hominimerdicola aceti*	80/3	2 585 427 614								
*Phocaeicola dorei*	175	642 979 370								643 123 629
*Phocaeicola vulgatus*	ATCC 8482	640 753 008								640 763 088
*Roseburia intestinalis*	L1‐82	2 562 617 159								
*Staphylococcus warneri*	NCTC7291	2 986 873 165		2 986 873 640			2 986 873 637			

^a^ The table shows the scientific name of the bacteria and the identification numbers of genes (IMG database) encoding potential NO_3_^−^ and NO_2_^−^ reductases. Amino acid sequences from biochemically well‐characterized enzymes were used as model proteins for a BLASTp analysis with the IMG database (https://img.jgi.doe.gov/cgi‐bin/m/main.cgi?section=FindGenomes&page=genomeSearch). Homologous proteins are indicated with the corresponding gene number. NasA: NAD(P)H‐dependent assimilatory NO_3_^−^ reductase from *K*. *oxytoca*; NarG: membrane‐bound respiratory nitrate reductase from *E. coli*; NapA: periplasmic respiratory nitrate reductase from *E. coli*; NrfA: periplasmic NO_2_^−^ reductase from *E. coli*; NirB: NADH‐dependent assimilatory NO_2_^−^ reductase from *E. coli*; NirS: NO‐forming NO_2_^−^ reductase from *P. stutzeri*; NirK: NO‐forming NO_2_^−^ reductase from *P*. *chlororaphis*; YtfE: Di‐Iron NO‐forming NO_2_^−^ reductase from *E. coli*.

### Analysis of nitrite degradation kinetics in the large intestine

The results shown in Table 1 were based on bioinformatic predictions. Therefore, it was necessary to verify the effects of the gut bacteria on the NO_2_^−^ content by measuring enzyme activities. To simulate the physiological conditions in the intestine, the nitrite content in the experiments needed to be set accordingly. The nitrite content was determined in fresh feces samples from five test subjects. Immediately after collecting 0.1 g of feces, samples were centrifuged (2 min, 1000 ***g***), and the supernatant was tested for nitrite content. No nitrite could be detected in the samples of the test subjects examined in this project. Data in the literature on the nitrite content vary widely, ranging from 0 μm to 400 μm. The mean value, 100 μm, was used as the physiological concentration in this study. Bacterial species from the large intestine (Table 2) were grown under a N_2_/CO_2_ atmosphere in buffered BHI medium (pH 7.0) with nitrite at 37 °C, and samples were taken regularly to measure the optical density, pH, and nitrite concentration. A simultaneous control without cells or inactivated cells was included to eliminate the possibility of nonspecific degradation.

**Table 2:** Kinetics of the nitrite consumption of highly abundant bacteria from the large intestine.

Species^a^	Nitrite degradation μmol g_DW_^−1^ h^−1^	Prevalence^b^	Abundance^c^
*Agathobacter rectalis*	< 1	88.0	6.2
*Akkermansia muciniphila*	< 1	44.5	0.7
*Bacteroides cellulosilyticus*	26.9 ± 12.2	51.3	3.4
*Bacteroides xylanisolvens*	110.4 ± 27.5	73.3	4.9
*Bifidobacterium longum*	2.2 ± 0.2	74.7	0.7
*Escherichia coli* K‐12	10 770 ± 2183	64.2	0.8
*Faecalibacterium prausnitzii*	< 2	86.8	3.5
*Hominimerdicola aceti*	< 1	65.0	2.5
*Phocaeicola dorei*	10.6 ± 6.2	69.1	17.4
*Phocaeicola vulgatus*	55.3 ± 32.2	90.3	15.0
*Roseburia intestinalis*	29.3 ± 10.9	67.1	1.1

^a^ Control assays with inactivated cells showed no nitrite reductase activity.

^b^ Blanco‐Míguez *et al*. [12].

^c^ King *et al*. [13].

From these data, the kinetics of nitrite degradation could be determined. For this purpose, the functional equation when plotting the NO_2_^−^ concentration over time was determined using the SciDAVis program (https://scidavis.sourceforge.net/) and the derivation of this function was calculated using a web tool (https://www.ableitungsrechner.net/). The data could then be used to determine the NO_2_^−^ decrease per time versus the optical density. The initial linear portions of the conversion rate curves were used to calculate the rate of nitrite degradation per g dry weight (g_DW_). This approach allowed for a comparison of the turnover rates of the gut bacteria, despite different growth rates (Table 2).

**Fig. 1:**
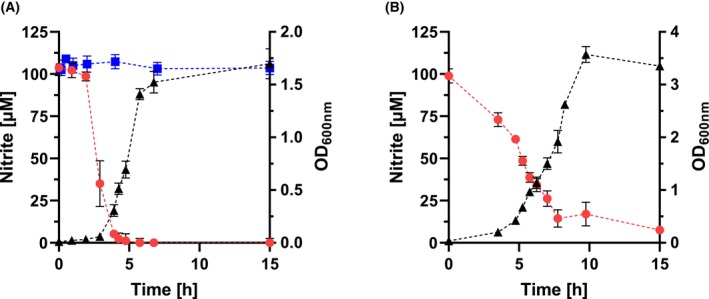
Nitrite degradation and growth of *E. coli* and *B. xylanisolvens*. A shows the data for *E. coli* and B contains the results for *B. xylanisolvens*. The initial NO_2_^−^ concentration was 150 μm and was corrected for the effect of cysteine to obtain a final NO_2_^−^ concentration of 100 μm. Cells were grown in buffered BHI medium under an N_2_/CO_2_ atmosphere at 37 °C. Samples were taken to analyze the optical density (OD_600_), the nitrite concentration, and the pH value (data not shown) as indicated. Black triangles, optical density; red circles, nitrite concentration; blue squares, nitrite concentration in medium without cells. Data represent mean ± SD from three independent biological experiments, each with three technical replicates.

*Escherichia coli* strains possess several NO_2_^−^ reductases, which are only produced under anoxic conditions and allow the reduction of nitrite to ammonia during anaerobic respiration. This reaction was also observed in our experiments, where even small amounts of cells were sufficient to completely reduce NO_2_^−^ (Fig. 1A). The pH remained stable during the first 5 h, indicating that a chemical degradation of nitrite in this period did not occur (data not shown). This reaction could not be observed in assays with heat‐inactivated *E. coli* cells and in experiments with only the cell‐free growth medium. Thus, it was clearly demonstrated that NO_2_^−^ degradation was enzymatically catalyzed and affected by chemical reactions. Overall, *E. coli* showed a very high activity, which was in the range of 11 000 μmol g_DW_^−1^ h^−1^ (Table 2). Enzyme‐catalyzed reduction of nitrite was also observed in *Bacteroides* (*B*.) *xylanisolvens* (Fig. 1B) and *B*. *cellulosilyticus* (data not shown). Like *E. coli*, these microbes also possess genes encoding the NrfA type nitrite reductase. However, the activity of these organisms ranged only from 27 to 110 μmol g_DW_^−1^ h^−1^, respectively (Table 2).

**Fig. 2:**
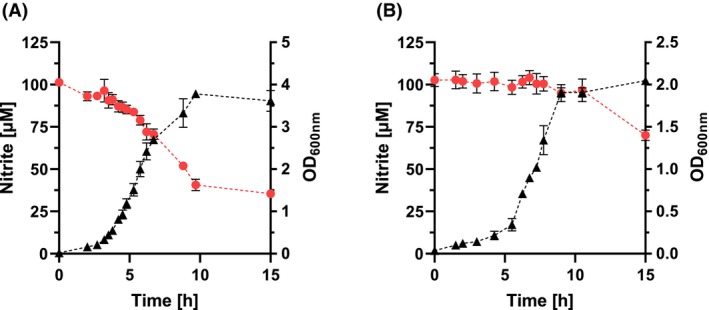
Nitrite degradation and growth of *P. dorei* and *B. longum*. The data for *P. dorei* and *B. longum* are shown in A and B, respectively. Cells were grown on buffered BHI with a final concentration of 100 μm nitrite under an N_2_/CO_2_ atmosphere at 37 °C. Samples were taken to analyze the optical density (OD_600_), the nitrite concentration, and the pH value (data not shown) as indicated. Black triangles, optical density; red circles, nitrite concentration. Data points represent mean ± SD from three experiments with three technical replicates each.

A nitrite reductase activity of 10.6 μmol g_DW_^−1^ h^−1^was also measured in *Phocaeicola* (*P*.) *dorei*, which was probably due to the NO‐producing enzyme YtfE (Fig. 2A, Table 2), an enzyme which is formed during nitrosative stress. Except for *Roseburia* (*R*.) *intestinalis*, all other colon bacteria without genes encoding nitrite reductases showed very low or no measurable enzymatic activity for reducing NO_2_^−^ (Tables 1 and 2). These organisms showed a slight linear reduction of the NO_2_^−^ concentration in the stationary phase without any correlation to the optical density or cell mass (e.g., *Bifidobacterium longum*, Fig. 2B). It is possible that some bacteria lysed in the stationary phase and intracellular compounds were released into the medium. Because nitrite can react with a variety of endogenous organic compounds, ranging from polyunsaturated fatty acids to tocopherols and glutathione, even at slightly acidic or neutral pH values, a reaction with these compounds cannot be excluded [14, 15, 16].

**Fig. 3:**
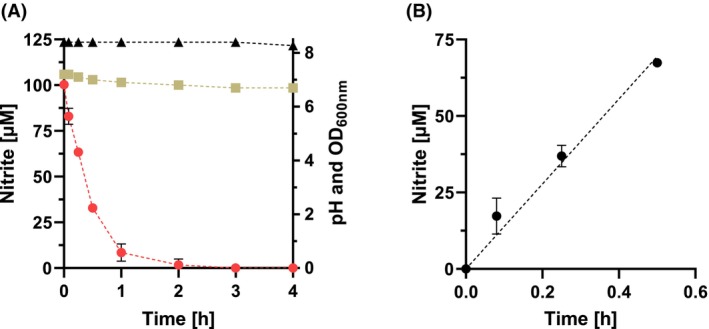
Nitrite degradation by the synthetic gut microbiota and kinetics of the reaction. A and B show the NO_2_^−^ degradation and the kinetics of the reaction, respectively. The strains were grown independently and mixed according to the relative abundance described by King *et al*. [13]. Resting but metabolically active cells with an OD_600_ of 8 in the buffered SHIME® salt solution were incubated at 37 °C under an anaerobic N_2_/CO_2_ atmosphere. The initial NO_2_^−^ concentration was 150 μm and was corrected for the effect of cysteine (materials and methods). (A) Red circles, nitrite concentration; brown squares, pH; black triangles, OD_600_. (B) Black circles, nitrite degradation. Data represent mean ± SD from three independent biological experiments, each with three technical replicates.

To simulate the conditions in the colon, preparations containing all intestinal bacteria together were also studied. The strains were grown individually and then mixed according to their abundance in the intestine (Table 2). This mixture of colon bacteria represents a synthetic bacterial biomatrix covering the most dominant genera identified in the human gut [13]. The preparation consisted of resting cells suspended in the buffered salt solution of the SHIME® medium [17]. Therefore, the medium did not contain any growth substrates. Due to storage conditions, these cells retained their full metabolic capacity for hours. This was evident from the stable optical density and cell numbers (Fig. 3), indicating that significant lysis did not occur. Experiments with growth media were also conducted, but it turned out that *E. coli* overgrew the inoculum very rapidly. Therefore, experiments with growing cells could not be performed. As evident from Fig. 3, the synthetic gut microbiota almost completely degraded NO_2_^−^ within 1 h. The highest activity was 70 μmol g_DW_^−1^ h^−1^. The NO_2_^−^ reducing activity of the gut mix was in the same range as the activity of *E. coli* (with an abundance of 0.8% in the colon), indicating that this organism (and related Enterobacteriaceae) might be of major importance for the detoxification of NO_2_^−^ in the large intestine.

**Fig. 4:**
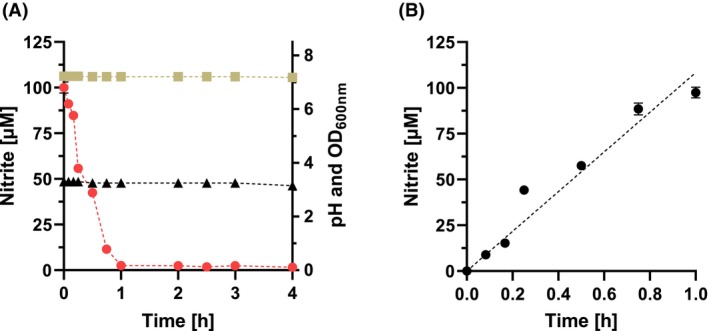
Nitrite reduction by fecal sample preparations and kinetics. (A) Nitrite degradation, pH variation, and OD_600_. (B) Nitrite degradation kinetics for fecal microbiota preparations. The microbiota was isolated by filtration from diluted fecal samples in buffered SHIME® salt solution. The cells were incubated anaerobically in an N_2_/CO_2_ atmosphere at 37 °C with an optical density (OD_600_) of 4 and effective 100 μm nitrite. (A) Red circles, nitrite concentration; brown squares, pH; black triangles, OD_600_. (B) Black circles, nitrite degradation. Data represent mean ± SD from three independent biological experiments, each with three technical replicates.

To further validate the previously obtained results under physiological conditions, the nitrite degradation kinetics of gut‐derived bacteria were analyzed. For this approach, the entire bacterial population was isolated from fecal samples that had been diluted in buffered SHIME® saline solution. The isolated fecal microbiota degraded nitrite within 1 h with maximum activities of about 100 μmol g_DW_^−1^ h^−1^ (Fig. 4). Overall, the reductive potential was determined for five human subjects, each with three technical replicates, resulting in an average nitrite degradation of 74.4 ± 24.5 μmol g_DW_^−1^ h^−1^.

### Analysis of nitrite degradation kinetics in the small intestine

The composition of the microbiota in the human small intestine is less well studied than the bacterial population in the colon. However, literature reports indicate that representatives of the genera *Lactobacillus*, *Limosilactobacillus* (*L*.), *Streptococcus* (*S*.), and *Veillonella* (*V*.) are highly abundant in the small intestine [18, 19, 20]. Therefore, the species *Lacticaseibacillus casei*, *L. reuteri*, *L. balticus*, *L. fermentum*, *S. vestibularis*, *S. hyointestinalis*, *S. thermophilus*, *V. magna*, *V. ratti*, and *V. atypica* were analyzed in this study. Over the course of these experiments, it became clear that most of the intestinal bacteria used in this study degraded nitrite within 24 h of incubation at 37 °C under anaerobic conditions. The kinetics of this reaction were then analyzed in detail. It was found that all species of the genera *Limosilactobacillus* and *Veillonella* degraded nitrite very rapidly with rates of 327 ± 28 to 3529 ± 255 μmol g_DW_^−1^ h^−1^, respectively (Table 3). These rates ranged from 3% to 33% in comparison to the degradation capacity of *E. coli*, respectively, and were much higher than the nitrite reducing activities of other representatives of the colon (*B. cellulosilyticus*, *B. xylanisolvens*, and *P. dorei*). High nitrite conversion rates were also determined for *S. vestibularis* (420 ± 122 μmol g_DW_^−1^ h^−1^). Interestingly, according to annotation databases, all these organisms do not possess genes that encode known nitrite reductases. In contrast, nitrite reductase activity was absent from *Lacticaseibacillus casei*, *S. hyointestinalis*, *and S. thermophilus* (Table 3).

**Table 3:** Nitrite reducing activities in lactic acid bacteria and species of the genera *Streptococcus* and *Veillonella*.

Species^a^	Nitrite degradation μmol g_DW_^−1^ h^−1^
*Lacticaseibacillus casei*	< 2
*Limosilactobacillus balticus*	890 ± 167
*Limosilactobacillus fermentum*	3529 ± 255
*Limosilactobacillus reuteri*	1070 ± 471
*Streptococcus hyointestinalis*	< 2
*Streptococcus thermophilus*	< 2
*Streptococcus vestibularis*	420 ± 122
*Veillonella atypica*	327 ± 28
*Veillonella magna*	771 ± 232
*Veillonella ratti*	922 ± 53

^a^ Cells were grown in the presence of an effective concentration of 100 μm nitrite under an N_2_/CO_2_ atmosphere at 37 °C. Samples were taken to analyze the optical density (OD_600_), the nitrite concentration, and the pH value (data not shown). The pH value of 7.0 did not change over the entire experiment. The activities represent results from three experiments with three technical replicates each.

### Impact of nitrite on bacterial growth

**Fig. 5:**
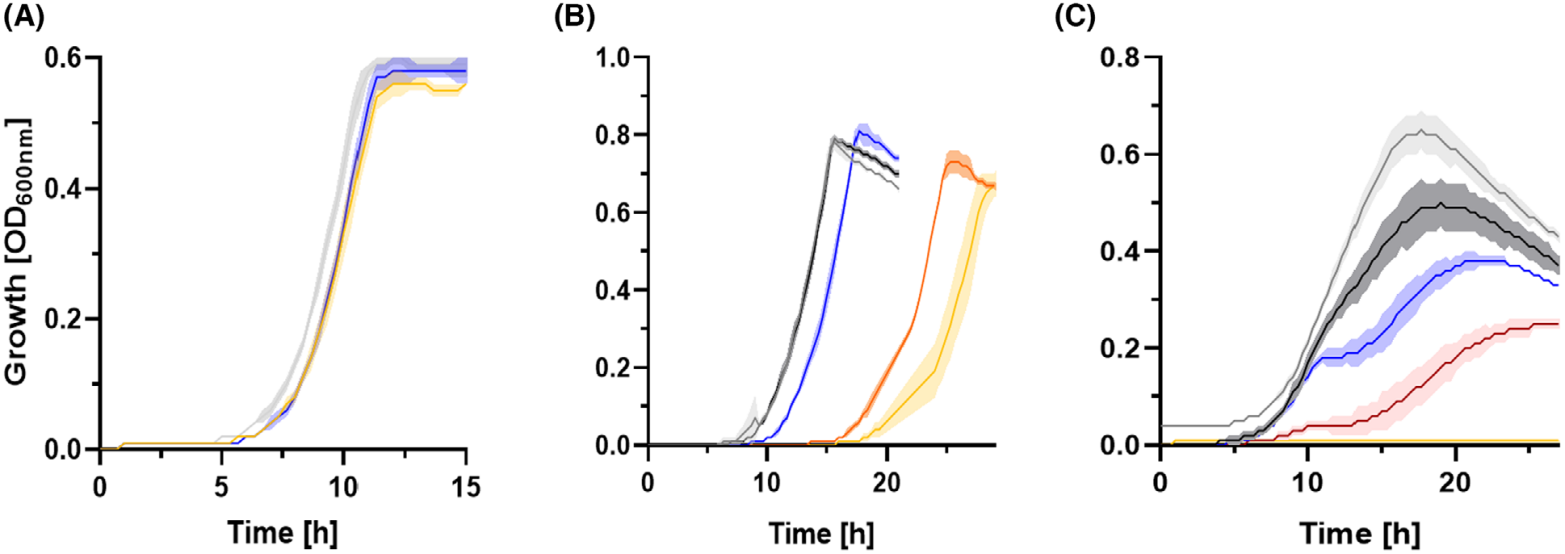
Nitrite‐dependent growth inhibition in *V. atypica*, *A. rectalis*, and *F*. *prausnitzii*. The figure shows the growth inhibition profiles of *V*. *atypica* (A), *A*. *rectalis* (B), and *F*. *prausnitzii* (C) in response to increasing nitrite concentrations. Strains were cultivated in Greiner CELLSTAR® 48‐well suspension culture plates with lid using a Tecan Infinite M200 plate reader (Tecan Group AG, Männedorf, Switzerland). During growth experiments, organisms were grown at 37 °C for 24 h under anaerobic conditions in an anoxic chamber (Coy Laboratory Products, Inc., Grass Lake, MI, USA) and maintained in a CO_2_/N_2_/H_2_ (49%/49%/2%) atmosphere. Key parameters such as optical density and pH were recorded. The central lines of the growth curves reflect the mean values of each biological triplicate, while the shaded area above and below indicates the standard deviation. Final nitrite concentrations: 0 μm (gray), 50 μm (black), 100 μm (blue), 200 μm (red), 500 μm (orange), 1 mm (yellow).

Interestingly, a nitrite concentration‐dependent inhibition of bacterial growth was observed for some species, indicating that nitrite plays a crucial role in regulating microbiota composition (Fig. 5). *V. atypica* was used as an example of a gut bacterium with high nitrite reductase activity. As expected, no significant changes in growth parameters were observed under varying nitrite concentrations. The cell density reached a maximum of 0.6 ± 0.05, with a lag phase of 5 h and a doubling time of 1.1 ± 0.16 h, indicating that *V. atypica* is able to reduce nitrite efficiently without any inhibitory effects on growth (Fig. 5A). *A. rectalis* showed an inconsistent growth behavior. The doubling time increased gradually from 0.74 ± 0.15 h at 0 μm nitrite to 1.74 ± 0.08 h at 1 mm nitrite. At the same time, the lag phase increased from 9 to 20 h, while the highest optical density remained unaffected at 0.75 ± 0.04. This shows that *A. rectalis* grew more slowly with an increased lag phase at higher nitrite concentrations but still achieved a similar maximum cell mass as observed under nitrite‐free conditions (Fig. 5B). A different type of growth inhibition was observed in *Faecalibacterium* (*F*.)* prausnitzii* (Fig. 5C). Up to a nitrite concentration of 200 μm, both the doubling time and lag phase remained unchanged at 0.80 ± 0.01 and 7.5 h, respectively. However, the maximum cell density gradually decreased with increasing nitrite concentration from 0.7 at 0 μm to 0.25 at 200 μm nitrite. Growth was completely inhibited at higher nitrite concentrations. These results indicated that *F. prausnitzii* was particularly sensitive to increasing nitrite concentrations, resulting in a significant impairment of growth. In summary, three different growth behaviors were identified in the strains without significant nitrite reducing activity: no inhibition (e.g., *B. longum*), extended lag phase (e.g., *A. rectalis*), and growth inhibition (*F. prausnitzii*, *Akkermansia muciniphila* (data not shown), *Hominimerdicola* (*H*.) *aceti* (data not shown)). Hence, the results demonstrate that nitrite had multiple effects on the growth of different gut bacterial strains and can thus substantially influence the composition of the gut microbiota.

### Reduction of nitrate

Pharmacology studies indicated that after ingestion, nitrate is rapidly absorbed in the proximal section of the small intestine, migrates into body fluids, such as saliva, and is finally excreted through urine [21]. It is suggested that only a small part of nitrate escapes absorption from the small intestine and is exposed to bacterial metabolism in the colon. Therefore, the question arose as to which gut bacteria are capable of reducing nitrate.

**Fig. 6:**
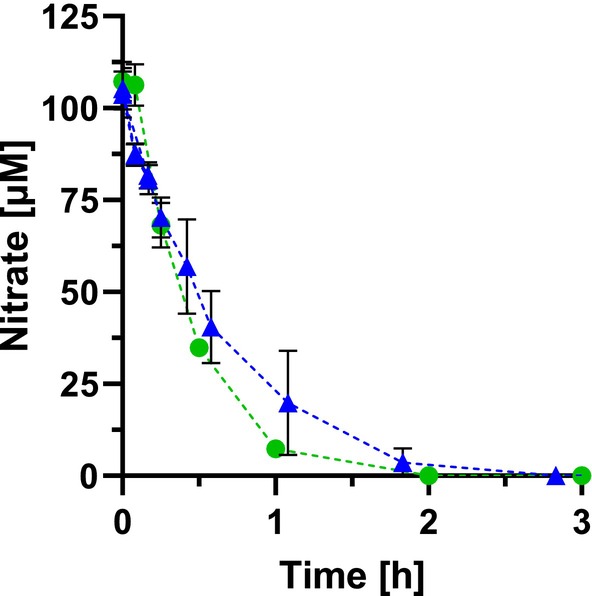
Nitrate degradation by the synthetic colon mixture and isolated fecal bacteria. The fecal microbiota was obtained by filtering diluted fecal samples in buffered SHIME® salt solution (green circles). For the preparation of the synthetic colon mixture, the strains (Table 7) were grown independently and mixed according to the relative abundance described by King *et al*. [13] (blue triangles). The assays were then performed at 37 °C under an anaerobic atmosphere of N_2_/CO_2_, with OD_600nm_ set to 2.1 (fecal bacteria) and 10.8 (colon mixture), along with a final nitrate concentration of 100 μm. The parameters pH, nitrite levels, and OD_600nm_ were regularly monitored. Data represent mean ± SD from three independent biological experiments, each with three technical replicates.

Most of our test organisms did not reduce NO_3_^−^ to NO_2_^−^ (Table 4), indicating that nitrate reductase activity was absent in these gut bacteria. This was likely due to either a lack of corresponding genes in their genomes (*A. muciniphila*, *B. longum*, *B. cellulosilyticus*, *P. dorei*, *B. xylanisolvens*, *P. vulgatus*, *R. intestinalis*, *F. prausnitzii*, and *A. rectalis*), or because the relevant genes were not expressed (*L. reuteri*). There were only two exceptions. *E. coli* reduced nitrate to nitrite with a rate of 487 μmol g_DW_^−1^ h^−1^. *V. atypica*, as a member of the small intestine microbiota, also showed NO_3_^−^ reducing activity with a rate of 208 μmol g_DW_^−1^ h^−1^ (Table 4). Both organisms possess NO_3_^−^ reductases. The genome of *E. coli* contains the genes for the expression of NarG, NapA, and NrfA type reductases. In *V. atypica*, the operon for the production of a NarG‐type NO_3_^−^ reductase was found. As in the experiments on nitrite degradation, the synthetic colon microbiota, which includes bacteria in proportions reflecting their abundance in the intestine (Table 2), as well as the microbiota isolated from fecal samples, were used in the study of nitrate reduction (Fig. 6). The synthetic bacterial mixture converted nitrate at a rate of 47.1 ± 2.9 μmol g_DW_^−1^ h^−1^, while the reduction rate of the isolated microbiota was 155.8 ± 46.8 μmol g_DW_^−1^ h^−1^. It can be assumed that all nitrate reduction originated from *E. coli* and relatives because all other species of the large intestine did not show nitrate degradation. As already demonstrated in the previous chapter for nitrite, *E. coli* and its close relatives (Enterobacteriaceae: e.g., species of the genera *Klebsiella*, *Salmonella*, *Citrobacter*, etc.) are of crucial importance for the nitrate metabolism in the colon. The anaerobic respiratory chains with nitrite and nitrate reductases at the end of the membrane‐bound electron transport systems are obviously highly efficient, allowing these organisms to persist in this environment. Despite its moderate abundance of only 0.8%, *E. coli* has a decisive influence on the nitrogen metabolism in the colon.

**Table 4:** Kinetics of the nitrate consumption of bacteria from the human intestinal tract.

Species	Intestine	Nitrate degradation μmol g_DW_^−1^ h^−1^
*Agathobacter rectalis*	Large	0
*Akkermansia muciniphila*	Large	0
*Bacteroides cellulosilyticus*	Large	0
*Bacteroides xylanisolvens*	Large	0
*Bifidobacterium longum*	Large	0
*Escherichia coli* K‐12	Large	487 ± 177
*Faecalibacterium prausnitzii*	Large	0
*Phocaeicola dorei*	Large	0
*Phocaeicola vulgatus*	Large	0
*Roseburia intestinalis*	Large	0
*Limosilactobacillus reuteri*	Small	0
*Streptococcus vestibularis*	Small	0
*Veillonella atypica*	Small	208 ± 52

## Discussion

NO_3_^−^ is present in our everyday diet, which represents a source of nitrogen as this anion is abundant in certain food products [22]. The oral cavity plays a central role in the initial bioactivation of dietary nitrate. It is a critical part of the nitrate–nitrite–nitric oxide (NO) pathway, which has emerged as a key axis linking the microbiome to host physiology. Salivary nitrite levels rise rapidly following nitrate intake, indicating the potential of oral nitrate‐reducing activity through bacteria, including species of the genera *Rothia*, *Neisseria*, *Actinomyces*, *Veillonella*, and *Propionibacterium* [23]. In the bloodstream, circulating nitrate is actively taken up by the salivary glands, concentrated, and again excreted in the saliva. As already mentioned, commensal facultative anaerobic bacteria convert nitrate to nitrite by the action of nitrate reductases [24]. The process is referred to as the enterosalivary circulation [25]. Once nitrite reaches the systemic circulation, a number of proteins and enzymes in blood and tissues can catalyze the one‐electron reduction of nitrite to nitric oxide (NO) [22]. NO serves as a signaling molecule in animals and humans acting as physiological mediators of cell‐to‐cell signaling in the regulation of cardiovascular function, immunity, metabolism, and more [26]. While the enterosalivary circulation of nitrate is well established, organ‐level distribution and excretion pathways remain less understood. It has been shown that intravenously administered nitrate in pigs is actively secreted into the proximal small intestine, where it accumulates and is then further reduced to nitrite [27].

In summary, NO_2_^−^ is either taken up and reduced to NO by human oxidoreductases, or is swallowed and quickly reaches the stomach through the esophagus. Concentrations of 77–520 μm nitrate were detected in human gastric juice [28, 29]. Because of the instability under acidic pH values (pH 1–4), direct measurements of nitrite in the gastric stage are hard to perform. At higher pH values, ranging from pH 5 to 8, nitrite was found at approximately 50–62 μm in the stomach [30]. Kondo *et al*. [31] determined a nitrite concentration in the gastric juice at a pH of 2.6 with 14 μm. Xu and Reed [32] detected nitrite concentrations of 0–650 μm depending on the pH value in the stomach (54 μm at pH 7). Nitrite, which significantly correlates with intragastric pH, is partly converted into NO in a reaction prompted by ascorbic acid in the acidic environment of the stomach [33, 34]. The remaining dietary nitrate and nitrite are absorbed early in the upper GI tract, followed by excretion of 75% of absorbed nitrate in the urine. There was no significant nitrite in the urine or ileal effluent. In accordance, nitrate concentrations in ileal effluent were between 30 and 60 μm. However, the possibility of significant amounts of nitrate and nitrite reaching the colon via the blood system in normal subjects is highly possible [35]. For people who consume a large amount of red meat, a second mechanism for the transport of nitrite to the colon is relevant. This is managed by heme‐mediated transportation, which begins with acid‐catalyzed thionitrosation (cysteine‐NO) of digested proteins in the stomach and the heme protein (Fe‐NO) itself [36, 37]. NO‐carrying proteins that reach the large intestine might be associated with oxidation to nitrite in the colon [38]. Although the precise mechanism remains unknown, close contact between fecal nitrosyl heme and oxygen from the colon's aerobic inner mucous layer is important for this process [39]. In line with this hypothesis is the fact that nitrite, as a potent nitrosating agent, is actually present in the contents of the feces of people with a high intake of red meat, compared to those with diets of white meat, fish, and vegetables [4].

Our experiments using single bacterial strains clearly showed that *E. coli* has a very high NO_2_^−^ reductase activity. However, this must be seen in context to the moderate abundance of the organism of about 0.8% in the colon of an average person [13]. Thus, with a bacterial count of 3.23 × 10^11^ bacteria per g of dry stool [40], there are 2.58 × 10^9^ *E. coli* cells per g of colon material. Considering the dry mass of one cell of about 2.52 × 10^−13^ g cell^−1^ (Table 5 [41, 42]), this results in a value of 0.65 mg_DW_ g_dry stool_^−1^ in the large intestine. The total stool content of the large intestine is estimated to be 400 mL [40], resulting in 104 g dry weight when a water content of about 75% is considered [43]. These values result in a DW of 68 mg for *E. coli* in this part of the human digestive tract. The NO_2_^−^ reductase activity of *E. coli* was 10 777 μmol NO_2_^−^ h^−1^ g_DW_^−1^ (Table 2), and thus, the total activity in the colon is in the range of 750 μmol h^−1^. These are merely numbers, but what do they mean for nitrite metabolism in the human body? A realistic scenario would be that a stool bolus with a NO_2_^−^ concentration of 50 μmol L^−1^ could reach the colon by food intake or NO_2_^−^ excretion from the human body. Assuming a feces volume of 400 mL (with stool density 1.04 g mL^−1^ [40]), this would correspond to an amount of 20 μmol NO_2_^−^ present in the total colon. Hence, the reduction of the total nitrite content in the colon by *E. coli* would require less than 2 min, indicating that even a small number of *E. coli* cells are able to completely detoxify nitrite in the colon in a very short time period. In summary, our experiments show that non‐pathogenic *E. coli* strains, as commensal inhabitants of the colon, have an important function in the detoxification of nitrite, which can be described as a probiotic property. In this context, we also refer to strain *E. coli* Nissle, which has long been classified as a probiotic strain [44]. The ability to reduce the NO_2_^−^ content of the human large intestine may contribute to the probiotic effects of this bacterium. In addition, there is a large number of *Bacteroides* and *Phocaeicola* species in the intestine, which have only a low nitrite reductase activity but are still relevant due to the large number of cells and account for approximately 10% of the total nitrite reducing activity.

**Table 5:** Calculation of the dry weight of one intestinal bacterium.

Organism	No. cells (L^−1^ OD1^−1^)	DW (g L^−1^ OD1^−1^)	DW one cell (g)
*Agathobacter rectalis*	1.85E+12	0.41	2.19E‐13
*Akkermansia muciniphila*	2.55E+12	0.41	1.62E‐13
*Anaerostipes hadrus*	6.95E+11		
*Bacteroides cellulosilyticus*	1.14E+12	0.31	2.72E‐13
*Bacteroides xylanisolvens*	2.23E+12	0.36	1.61E‐13
*Bifidobacterium adolescentis*	1.41E+12	0.42	2.98E‐13
*Blautia faecis*	1.69E+12	0.45	2.63E‐13
*Escherichia coli*	8.00E+11	0.22	2.80E‐13
*Faecalibacterium prausnitzii*	1.17E+12	0.41	3.51E‐13
*Mediterraneibacter gnavus*	9.78E+11	0.25	2.56E‐13
*Parabacteroides johnsonii*	1.52E+12	0.40	2.63E‐13
*Phocaelicola dorei*	1.61E+12	0.23	1.43E‐13
*Phocaelicola vulgatus*	1.03E+12	0.36	3.50E‐13
*Segatella copri*	1.44E+12	0.40	2.78E‐13
Average	1.43E+12	0.36	2.52E‐13
StDV	5.28E+11	0.08	6.64E‐14

The total nitrite reduction activity can also be calculated using the data measured for the feces samples and the parameters described above. Based on a bacterial count of 3.23 × 10^11^ cells g_dry stool_^−1^, this results in a dry weight of 81 mg mL^−1^ stool (Table 6) which corresponds to the values given by Rose *et al*. [43]. The specific activity of the stool samples was 74.4 μmol g_DW_^−1^ h^−1^. This results in a total activity of 624 μmol h^−1^ (for a dry stool content of 104 g; average of five probands), indicating that the abundance of *E. coli* was slightly lower than calculated by King *et al*. [13] and was probably in the range of 0.7%. However, the measured and calculated activities were in the same range, indicating that the parameters used for the calculations were in the correct frame. Overall, the activity was so high that the nitrite concentration in the human stool should be far below our measurement sensitivity of 10 μm. This statement turned out to be correct because we could not detect nitrite in our tested feces samples. This low value is also necessary to allow nitrite‐sensitive microorganisms to colonize the intestine. Otherwise, important groups of bacteria such as *A. rectalis*, *F. prausnitzii*, and *Akkermansia muciniphila* would be prevented from growing (Fig. 5), which would lead to a pathological shift in the composition of the intestinal microbiota.

**Table 6:** Calculation of nitrite reductase activity in the colon.

Parameter	*Escherichia coli* (0.8% in colon)^a^	Feces
Bacterial cells (g_dry stool_^−1^)	2.58 × 10^9^	3.23 × 10^11^ ^c^
Dry mass one bacterial cell (g_DW_)^b^	2.52 × 10^−13^	2.52 × 10^−13^
Bacterial cell mass (g_DW_ g_dry stool_^−1^)	0.00065	0.081
Specific activity (μmol g_DW_^−1^ h^−1^)	10 966	74.4
Dry stool activity (μmol g_dry stool_^−1^ h^−1^)	7.1	6.0
Colon content (mL_wet stool_)^d^	400	400
Colon content (g_wet stool_)^d^	416	416
Colon content (g_dry stool_)^e^	104	104
Total activity colon (μmol h^−1^)	742	624

^a^ Average abundance of *E. coli* in feces samples (0.8%) [13].

^b^ Dry weight of one gut bacterium = 2.52 × 10^−13^ g (Table 5).

^c^ Number of bacteria in 1 g_DW_ feces = 3.23 × 10^11^ g^−1^ [40].

^d^ Wet stool density 1.04 g mL^−1^ [40].

^e^ Wet stool has a H_2_O content of 75% [40, 43].

Our observation that NO_2_^−^ is rapidly reduced in the colon aligns with the majority of published studies dealing with the nitrate and nitrite metabolism in the gut microbiota. For example, it was shown that *E. coli* converts nitrate to nitrite and ammonium, and it is still a matter of debate whether NO is formed by *E. coli*. Strains of *L. rhamnosus*, *L. acidophilus*, and *Bifidobacterium longum infantis* grown with nitrate showed minor changes in nitrite or ammonia levels. However, when supplied with exogenous nitrite, NO gas was produced probably by a chemical mechanism due to a decrease in the pH value [45, 46].

For the large intestine, physiological nitrite concentrations ranging from 0–20 μm nitrite have been reported [4, 35, 37, 47, 48, 49, 50, 51]. In contrast, in one publication, very high nitrite concentrations of over 400 μm NO_2_^−^ were detected in human feces [52]. These values are difficult to explain because of the high nitrite reducing activity of the gut microbiota. In our study, nitrite concentrations were below the detection limit of 10 μm in all human fecal samples analyzed (data not shown).

In nitrite‐ammonifying bacteria, there may be two distinct pathways for converting nitrite to ammonia, each serving different physiological functions and utilizing enzymes with distinct catalytic centers [11]. The first process involves assimilatory or detoxifying enzymes (e.g., NasB from *K*. *oxytoca* or NirB from *E. coli* [53]) which feature a special porphyrin cofactor in close proximity to a [4Fe‐4S] cluster, known as the siroheme‐[4Fe‐4S] catalytic center. The second protein family comprises respiratory multiheme c‐type cytochromes, which contain multiple heme c groups (e.g., NrfA from *E. coli*). The third class of nitrite reductases is characterized by multiple copper centers involved in the catalytic cycle (e.g., NirK from *P*. *chlororaphis*). An additional family of nitrite reductases comprises heme cd1 proteins (e.g., NirS from *P*. *stutzeri*). The last class is the recently discovered di‐iron NO_2_^−^ reductases, which are involved in managing nitrosative stress conditions (e.g., YtfE from *E. coli*). While NasB and NrfA reduce nitrite to ammonium, NirK, NirS, and YtfE form nitric oxide from NO_2_^−^.

(1) $${{\mathrm{NO}}_{2}}^{-}+6\;e^{-}+8\;H^{+}\to {{\mathrm{NH}}_{4}}^{+}+2\;H_{2}O$$

(2) $${{\mathrm{NO}}_{2}}^{-}+e^{-}+2\;H^{+}\to \mathrm{NO}+H_{2}O$$

Our experiments clearly show that several gut bacteria were able to reduce nitrite. Bioinformatic analyses indicated that this activity could be assigned to known nitrite reductases (*E. coli*, NrfA, NirB, YtfE; *Bacteroides* species, NrfA, YtfE; *Phocaeicola* species, YtfE; *S*. *warneri*, NirB; Table 1). In contrast, intestinal bacteria without annotated nitrite reductases were indeed incapable of reducing the nitrite concentration by means of enzymes (Table 2). However, there were exceptions. For example, *R. intestinals* reduced nitrite without a corresponding enzyme being annotated. The same applied to microorganisms from the small intestine (Table 3). The ability of lactic acid bacteria to reduce nitrite has been documented for a long time [54]. For example, nitrite degradation has been described for *Lactobacillus viridescens*, *Lactobacillus acidophilus*, *Lactococcus lactis*, *Lacticaseibacillus rhamnosus*, *L. fermentum*, *Lactiplantibacillus plantarum*, *Leuconostoc mesenteroides*, and *Pediococcus pentosaceus* [54, 55, 56, 57]. The results of this work show significant nitrite reducing activity by *L. reuteri*, exceeding 1000 μmol g_DW_^−1^ h^−1^, although bioinformatic analyses did not indicate the presence of known nitrite reductases. Similar findings in the closely related species *L. balticus* and *L. fermentum* also suggest that this could potentially be a genus‐specific characteristic (Table 3). However, the literature does not provide a consistent understanding of the degradation mechanisms within a species. The kinetics recorded in this study revealed an exponential decrease of nitrite/nitrate concentration, suggesting the involvement of enzymatic mechanisms. Chemical degradation by acidification could be ruled out as a strongly buffered medium was used and the pH value was maintained constant at ≥6. The ability to reduce nitrite also appears to vary within the genus *Streptococcus* and remains poorly understood in many aspects. While some *Streptococcus* species, including *S. sanguinis*, can reduce nitrite only under specific growth conditions, many other species have not been found to reduce nitrite at all [58]. Other members of the genus, including *S. mutans*, also have genes that are relevant for nitrogen metabolism, such as the nitrite reductase gene (*nirJ*) [59] and show the enzymatic reduction of nitrite to nitric oxide. In this study, nitrite reduction was detected in *S. vestibularis* at a rate exceeding 400 μmol g_DW_^−1^ h^−1^. In contrast, the closely related species *S. thermophilus* and *S. hyointestinalis* showed no measurable nitrite reducing activity (Table 3). This variability indicates that nitrite reduction is a specific characteristic of individual species and is not universally present across the entire genus.

Previous studies on nitrate–nitrite metabolism in the genus *Veillonella* have primarily focused on the reduction of nitrate to nitrite [60, 61], with less attention given to nitrite reduction. Although the first evidence of nitrite degradation was found for *V. alcalescens* [62], the underlying mechanism remained unclear. Later studies on *V. parvula* confirmed nitrite reduction, but only in very low activities (10 μmol g_DW_^−1^ h^−1^) [63]. In this study, we were able to show for the first time that several species of the genus *Veillonella*, including *V. atypica*, *V. magna*, and *V. ratti*, exhibit significant nitrite reduction (up to 900 μmol g_DW_^−1^ h^−1^), although there is no bioinformatic evidence for the presence of known nitrite reductases. These results indicate that *Veillonella* species are capable of nitrite reduction, but the enzymes potentially involved in this reduction have not yet been identified. Overall, however, the proportion of small intestine bacteria in nitrite detoxification is likely to be low, as the bacterial density (max. 10^8^ cells mL^−1^) is orders of magnitude lower than the bacterial population in the large intestine (up to 10^11^ cells mL^−1^).

In summary, our findings align with those of earlier studies that demonstrated the ability of selected gut bacteria to convert nitrate/nitrite to NH_4_^+^ or NO, although this capacity varies widely between species. For example, it was shown that *E. coli* readily reduces nitrite under anaerobic conditions, while *Bifidobacterium and Lactobacillus* primarily contribute to non‐enzymatic NO formation under acidic conditions [45, 46]. These studies further support our conclusion that enzymatic nitrite reduction in the colon is driven by a limited number of bacterial taxa, particularly *E. coli*, while broader nitric oxide generation likely depends on microbial composition and local environmental factors, such as pH.

## Materials and methods

### Materials

Media components and general laboratory reagents were obtained from Carl Roth GmbH (Karlsruhe, Germany), whereas substrates and vitamins were purchased from Sigma‐Aldrich (St. Louis, MO, USA). The gases CO_2_ (99.9%), H_2_ (99.9%), and N_2_ (99.9%) were obtained from Air Liquide (Düsseldorf, Germany).

### Bacterial strains and standard culture conditions

Strains were obtained from the German Collection of Microorganisms and Cell Cultures (DSMZ; Brunswick, Germany) and were grown anaerobically in complex brain heart infusion (BHI) medium buffered to pH 7.0 with 65 mm KHCO_3_ and 35 mm potassium phosphate. To establish anoxic conditions, the serum flasks used for cultivation were first flushed with an N_2_/CO_2_ gas mixture (80%/20%) and then sealed with butyl rubber stoppers. Before inoculation, the media were supplemented with hemin (5 mg L^−1^), vitamin K1 (1 μL L^−1^), a vitamin solution (1 mL L^−1^ [64]), and L‐cysteine (0.26 g L^−1^) as a reducing agent as well as 1 mg L^−1^ resazurin as a redox indicator. The cells were cultured at 37 °C, and their growth was monitored by measuring the optical density at 600 nm (OD_600_). To perform small‐scale growth experiments, liquid cultures were grown in 48‐well microtiter plates in a heatable plate reader (Tecan, Männedorf, Switzerland). Cultures were maintained under strictly anaerobic conditions in an anaerobic tent (Coy Laboratory Products Inc., Grass Lake, MI, USA) with an atmosphere made of N_2_ (78%), CO_2_ (20%), and H_2_ (2%). Data analysis was performed with Magellan 6 software (V6.5; Tecan, Männedorf, Switzerland).

### Selection of gut bacteria

The microbiota in the intestinal tract of humans is highly diverse and differs from individual to individual. More than 1000 species were detected in the human colon [12, 65], indicating that the number is too high to analyze all these bacteria separately. Several publications are available that describe a numerical analysis of the microbiota in the different organs of humans [13, 66, 67]. According to the “core species” concept [13], strains should be analyzed that are present in almost all individuals and in large quantities in the human colon. As a result, we have selected 21 highly abundant organisms (Table 7) that represent the most prominent phyla: Bacteroidota, Bacillota, and Pseudomonadota. In addition, one representative of each of the phyla Actinomycetota and Verrucomicrobia was added. The selection includes the most prevalent genera found in the human gut, such as *Akkermansia*, *Bacteroides*, *Phocaeicola*, *Eubacterium*, *Faecalibacterium*, *Ruminococcus*, *Escherichia*, *Roseburia*, and *Bifidobacterium*. Corresponding species were identified from comprehensive metagenomic taxonomic profiling studies [12, 13]. To simulate the conditions in the large intestine, a “gut mix” was established. For this purpose, the strains found in the large intestine (Table 7) were grown individually and then mixed according to their abundance in the intestine. This mixture of colon bacteria represents a synthetic bacterial biomatrix comprising approximately 60% of all prokaryotes found in an average human colon. Furthermore, this assortment includes the most dominant genera identified in the human gut. Consequently, these species act as representative organisms for their respective genera. Together, the selected genera account for approximately 83% of all colonic bacteria [13]. The composition of the microbiota in the human small intestine is less well studied than the bacterial population in the colon. However, data in the literature indicate that representatives of the genera *Lactobacillus*, *Streptococcus*, and *Veillonella* are present in high abundance [18, 19, 20]. Therefore, several species of these genera were analyzed in this study (Table 7).

**Table 7:** Gut bacteria used in this project.

	DSM No.	Phylum	Family
Species (large intestine)			
*Agathobacter rectalis* ^a^	17 629	Bacillota	Lachnospiraceae
*Akkermansia muciniphila* ^b^	22 959	Verrucomicrobia	Akkermansiaceae
*Bacteroides cellulosilyticus*	14 838	Bacteroidota	Bacteroidaceae
*Bacteroides xylanisolvens*	18 836	Bacteroidota	Bacteroidaceae
*Bifidobacterium longum*	20 088	Actinomycetota	Bifidobacteriaceae
*Escherichia coli*	6574	Proteobacteria	Enterobacteriaceae
*Faecalibacterium prausnitzii*	17 677	Bacillota	Oscillospiraceae
*Hominimerdicola aceti* ^c^, ^d^	102 216	Bacillota	Oscillospiraceae
*Phocaeicola dorei* ^e^	17 855	Bacteroidota	Bacteroidaceae
*Phocaeicola vulgatus* ^f^	1447	Bacteroidota	Bacteroidaceae
*Roseburia intestinalis*	14 610	Bacillota	Lachnospiraceae
Species (small intestine)			
*Lacticaseibacillus casei* ^g^	own isolate	Bacillota	Lactobacillaceae
*Limosilactobacillus balticus* ^h^	110 574	Bacillota	Lactobacillaceae
*Limosilactobacillus fermentum* ^i^	20 052	Bacillota	Lactobacillaceae
*Limosilactobacillus reuteri* ^j^	20 016	Bacillota	Lactobacillaceae
*Streptococcus hyointestinalis*	20 770	Bacillota	Streptococcaceae
*Streptococcus thermophilus*	20 617	Bacillota	Streptococcaceae
*Streptococcus vestibularis*	5636	Bacillota	Streptococcaceae
*Veillonella atypica* ^k^	20 739	Bacillota	Veillonellaceae
*Veillonella magna* ^k^	19 857	Bacillota	Veillonellaceae
*Veillonella ratti* ^k^	20 736	Bacillota	Veillonellaceae

^a^ Former *Eubacterium rectale*.

^b^ Supplemented with precipitated and purified mucin derived from 75 to 95% porcine gastric mucosa in the final concentration of 1.0% (w/v).

^c^ Former *Ruminococcus bicirculans*.

^d^ Growth with fatty acid mixture with the final concentration of 5 mm acetate, 5 mm propionate, 5 mm butyrate, 1 mm iso‐butyrate, 1 mm valerate, 1 mm iso‐valerate, and 1 mm 2‐methylbutyrate.

^e^ Former *Bacteroides dorei*.

^f^ Former *Bacteroides vulgatus*.

^g^ Former *Lactobacillus casei*.

^h^ Former *Lactobacillus balticus*.

^i^ Former *Lactobacillus fermentum*.

^j^ Former *Lactobacillus reuteri*.

^k^ Addition of D/L‐lactate mixture in the final concentration of 1.5% (w/v).

### Fecal sample collection and ethical approval

All procedures involving human participants were performed in accordance with the ethical standards of the institutional and national research committee and with the 1964 Declaration of Helsinki and its later amendments. Written informed consent was obtained from all individual participants included in the study. The study protocol was approved by the Ethics Committee of the University of Bonn (approval number: NCT06072391). Human fecal samples were collected at the Institute of Nutrition and Food Sciences, Life & Medical Sciences Institute, Rheinische Friedrich‐Wilhelms‐Universität Bonn, in January 2024.

### Bioinformatic prediction for the presence of genes encoding nitrite/nitrate reductases in gut bacteria

The genomes of 113 gut bacteria were checked for the presence of genes encoding nitrate reductases and nitrite reductases using the program BLASTp at IMG (https://img.jgi.doe.gov/cgi‐bin/m/main.cgi?section=FindGenomes&page=genomeSearch) with default parameters and a threshold e‐value of 1e^−40^ (Table S1). A collection of well‐characterized enzymes was used as reference (NasA, Q06457, assimilatory nitrate reductase from *K*. *oxytoca*; NarG, BAA36094.1, respiratory nitrate reductase 1, alpha subunit from *E. coli* K‐12; NapA, BAA15989.2, periplasmic nitrate reductase, large subunit from *E. coli* K‐12; NasB, Q06458, assimilatory nitrite reductase [NAD(P)H], large subunit from *K*. *oxytoca*; NrfA, P0ABK9, periplasmic penta‐heme cytochrome c nitrite reductase from *E. coli* K‐12; NirK, Q06006, copper‐containing nitrite reductase from *P*. *chlororaphis* (*aerofaciens*); NirS, P24040, nitrite reductase from *P*. *stutzeri*; YtfE, AAC77166.1, di‐iron nitrite reductase from *E. coli* K‐12). All nitrate reductases form nitrite from nitrate. NapA and NasB produce ammonia from nitrite. NirK and YtfE reduce nitrite to NO. NirS homologs were not found in any of the analyzed GIT bacteria.

### Colorimetric nitrite quantification

Nitrite concentration was quantified colorimetrically using the Lunge Reagent method. The first reagent (SA solution) was prepared by dissolving 1 g of sulfanilic acid in 30 mL of glacial acetic acid. Distilled water was added to a final volume of 100 mL. The second reagent (NA solution) was made by dissolving 1 g of 1‐naphthylamine in 50 mL of distilled water. Upon the addition of nitrite, the reaction of these two solutions produces an azo dye with an absorption peak at 540 nm [68]. Calibration was performed using a nitrite standard curve (0–100 μm Na‐nitrite) prepared in BHI medium, with absorbance measured at 540 nm. To determine the nitrite concentration, 50 μL of the sample and one positive and one negative control, each with three technical replicates, were transferred to a 96‐well plate and mixed with 50 μL SA solution and 50 μL NA solution. After an incubation time of 10 min, the plate was centrifuged to remove suspended solids. The absorbance of the supernatant was measured at 540 nm, and the nitrite concentrations were determined using a calibration curve.

### Quantification of nitrate and nitrite using HPLC

The quantification of nitrate and nitrite was carried out using a Knauer HPLC system (Knauer GmbH, Berlin, Germany), which included a degasser (Knauer Smartline Manager 5000), a pump (Knauer Smartline Pump 1000), an autosampler (Knauer Smartline Autosampler 3800), and a UV detector (Knauer Smartline UV Detector 2600). Sample separation was performed with the column LiChrospher® 100 RP‐18 endcapped (5 μm) and precolumn LiChrospher® 100 RP‐18 endcapped (5 μm) (Merck KGaA, Darmstadt, Germany) at 30 °C. The mobile phase, 0.01 m octylamine with pH 7.0, was used in isocratic mode with a flow rate of 0.8 m Lmin^−1^. To remove insoluble substances, the samples were treated with octylamine at a final concentration of 0.01 m and centrifuged (11 000 ***g***, 1 min) prior to HPLC analysis. A constant injection volume of 20 μL was ensured by using a suitable sample loop. Quantification was performed with external standards. Data analysis was performed with ClarityChrom (Knauer GmbH, Berlin, Germany).

### Reaction of NO_2_ ^−^ with growth media in the absence of bacteria

**Fig. 7:**
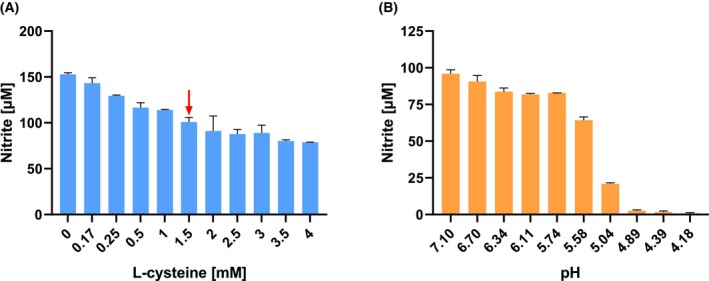
Effect of cysteine on the degradation of NO_2_^−^ after 24 h incubation and pH‐dependent decay of NO_2_^−^. (A) BHI medium with different cysteine concentrations was incubated for 24 h without the addition of bacteria under anaerobic conditions with a NO_2_^−^ concentration of 150 μm at 37 °C. Arrow, concentration of cysteine used in experiments. Samples were taken at different time points, and the concentration of NO_2_^−^ was determined. All measurements were performed with three technical replicates. (B) 100 μm NO_2_^−^ was added to 500 μL growth medium with the indicated pH value under anaerobic conditions at 37 °C for 24 h. All measurements were performed in triplicate. Data represent mean ± SD from three technical replicates.

In complex media, components, especially glutathione and unsaturated fatty acids as well as reducing agents such as cysteine, could be present that can chemically react with NO_2_^−^. Therefore, it was necessary to analyze whether the BHI medium used for the cultivation of the gut bacteria is involved in the degradation of NO_2_^−^ simply by chemical reactions. The medium was incubated for 24 h without the addition of bacteria under anaerobic conditions with a NO_2_^−^ concentration of 100 μm at 37 °C. Samples were taken at different time points, and the concentration of NO_2_^−^ was determined. The results showed that in BHI medium, the NO_2_^−^ concentration dropped to 50 μm within seconds and then stayed constant over 24 h (not shown). Hence, a significant amount of NO_2_^−^ reacted with media components chemically in the absence of bacteria. Different medium components were anaerobically incubated for 24 h at 37 °C in the presence of 150 μm NO_2_^−^ to investigate which compound led to the decrease of the NO_2_^−^ concentration. It became evident that the NO_2_^−^ content was not affected by complex media components (peptone or yeast extract) up to a concentration of 3 g L^−1^ (data not shown). In contrast, the NO_2_^−^ concentration was influenced by cysteine, which functions as oxygen protection in the growth medium (Fig. 7A). Under these conditions, the NO_2_^−^ concentration decreased almost linearly with increasing L‐cysteine concentrations down to about 80 μm after 24 h in the presence of 4 mm cysteine.

Because of these results, 1.5 mm cysteine was used in the assays, resulting in NO_2_^−^ concentration of 100 μm. In general, cysteine could not be omitted because no growth of the strictly anaerobic bacteria occurred without this reducing agent. Substitute reducing agents such as dithiothreitol, Na_2_S, or titanium (III)‐citrate had a more severe effect on NO_2_^−^ that was completely reduced after the addition of these alternative reducing agents.

All intestinal bacteria have a fermentative metabolism and produce large amounts of organic acid, which can lower the pH down to 4.0 over time. Hence, the pH of the growth media was also of crucial importance. At a pH value below 5.8, parts of the NO_2_^−^ were already scavenged chemically, resulting in the protonation of NO_2_^−^ to HNO_2_/NO^+^ and subsequent nitrosation of secondary amino groups or thiol groups present in the complex medium (Fig. 7B). In contrast, media with a pH >5.8 showed only a minor effect on the NO_2_^−^ concentration, which was reduced by about 10%. This finding led us to use strongly buffered BHI medium by adding 65 mm KHCO_3_ and 35 mm potassium phosphate to prevent a drop of the pH below 6.0.

### Nitrate and nitrite reduction assay by gut bacteria and feces

To assess the nitrite reducing capacity of the intestinal bacteria, the degradation was investigated kinetically. The different strains were anaerobically inoculated in serum bottles with 1.5 mm cysteine and 100 μm nitrate or 150 μm Na‐nitrite to reach an intestinal physiological level of 100 μm nitrite in the medium. The cultures were incubated at 37 °C for 24 h, with regular sampling to measure OD_600_, pH, and nitrite/nitrate concentration. As a control, the nitrite concentration was measured both in cell‐free buffer and in heat‐inactivated cells. For inactivation, the cells were incubated at 100 °C for 60 min and then cooled to room temperature. For nitrite measurement, 50 μL of culture was mixed with an equal volume of 99.8% acetonitrile and was frozen at −80 °C. The nitrite content was quantified colorimetrically, while nitrate was determined by HPLC.

To further simulate the physiological conditions in the intestine, the gut mix was analyzed. The selected strains were combined according to Table 2. To prevent a change in composition due to incubation at 37 °C, the cells were washed and resuspended in a modified SHIME® medium (29.8 mm NaHCO_3_, 34.2 mm NaCl, 18.7 mm NH_4_Cl, 49.5 mm K_2_HPO_4_, 49.8 mm KH_2_PO_4_, 0.07 mm CaCl_2_ × 2 H_2_O, 0.03 mm MgSO_4_ × 7 H_2_O, 1 mg L^−1^ resazurin) [17]. The cells no longer underwent any significant cell divisions but were metabolically active. For the nitrite degradation assay, a final OD_600_ of 10 was adjusted, and the cells were analyzed as described above. Furthermore, fresh feces samples were prepared and incubated in the presence of nitrite to compare the results with the data from the synthetic colon microbiota. For this, 5 g of fresh feces were filled into a 100 mL serum flask and diluted in 50 mL modified SHIME® medium containing 5 mm dithiothreitol as a reducing agent. Immediately, the flask was flushed with N_2_/CO_2_ to remove O_2_ from the flask atmosphere. All further purification steps were performed in an anaerobic hood. The samples were filtered, and the eluate was centrifuged at 500 x g to get rid of suspended particles. The samples were then centrifuged at high speed and washed twice with anaerobic buffer. In this way, all microorganisms were collected at the bottom of the centrifugation vial. Finally, the bacterial cell pellet was solved in SHIME® medium with 1.5 mm L‐cysteine to an optical density of 8–10, and kinetics were analyzed as described below.

### Determination of dry weight and cell number

To calculate the NO_2_^−^ reducing activities, it was important to quantify the reactions. Therefore, a reference point had to be found to calculate the DW of the intestinal bacteria in fecal samples. The starting point for this calculation was the number of microbes in a fecal sample, which was defined by Sender *et al*. [40] as 3.23 × 10^11^ cells g_dry stool_^−1^. It was then crucial to determine the average mass of an intestinal bacterium. For this purpose, different species from different genera and phyla were cultivated and harvested at a minimum of three different OD values (Table 5). The number of cells was determined with a Thoma counting chamber (Glaswarenfabrik Karl Hecht GmbH & Co KG, Sondheim, Germany), with a cell depth of 0.02 mm and an area of 0.0025 mm^2^. Cell counts were performed using an Olympus CX43 microscope (Evident GmbH, Hamburg, Germany). A total of four main squares was counted. The samples were washed, dried, and the DW was determined after 24 h and 48 h. The DW was plotted against the cell number, and the average values were calculated from the slope of the curves. Finally, the average cell mass from all species was calculated and resulted in a value of 2.52 × 10^−13^ g cell^−1^ (Table 5). This value represents an average mass of gut bacteria and was in accordance with other publications which indicated values of 3.0 × 10^−13^ and 2.8 × 10^−13^ g cell^−1^ [40, 69] and was used to calculate the bacterial dry weight in fecal samples.

## Conflict of interest

There is no conflict of interest for all authors.

## Author contributions

NH planned experiments, performed experiments, analyzed data, and wrote the paper. MG planned experiments, performed experiments, and analyzed data. MH contributed to the development of the analytical methodology. JLCMD provided methodological and scientific support. UD designed the study, planned experiments, analyzed data, wrote the paper, and supervised the study.

## Supporting information


**Table S1.** Presence of enzymes involved in nitrogen metabolism in 113 most important bacterial species in the colon.

## Data Availability

All raw data supporting the findings of this study have been deposited in the Dryad Digital Repository and are publicly available at: https://doi.org/10.5061/dryad.stqjq2cf4.
