# Supplementary material for: Distribution and activity of nitrate and nitrite reductases in the microbiota of the human intestinal tract
Source: FEBS J. 2025 Nov 5;293(6):1624–42. doi: 10.1111/febs.70299 (PMC12998184; doi:10.1111/febs.70299)
Supplement: Supplementary file 1 — Table S1. Presence of enzymes involved in nitrogen metabolism in 113 most important bacterial species in the colon. [file FEBS-293-1624-s001.pdf]

Suppl. Tab. 1: Presence of enzymes involved in nitrogen metabolism in 113 most important bacterial species in the colon. The genomes of all species were checked for the presence of genes encoding nitrate reductases (NasA, NarG, NapA) [a)] and nitrite reductases (NasB, NrfA, Nirk, YtfE) [a)], using the program BLASTp at the IMG database with a threshold e-value = 1e-40 (https://img.jgi.doe.gov/). Here shown are their identification number from the Human Metagenome Project (IMG No. column 2), taxonomic position (column 3), isolation location (column 4), and the results (columns 5-11).

Organism Name	Genome ID	Taxonomy (Phylum/ family)	Isolation	NasA	NarG	NapA	NasB	NrfA	NirK	YtfE
Acetanaerobacterium elongatum CGMCC 1.5012	2667527408	Bacillota; Oscillospiraceae	GIT							
Acidaminococcus sp. HPA0509	2541047012	Bacillota, Acidaminococcaceae	GIT							
Actinomyces sp. HPA0247	2541047011	Actinomycetota, Actinomycetaceae	GIT		2541441839					
Aeromonas media DSM 4881	2645727928	Pseudomonadota, Aeromonadaceae	GIT	2647589559		2647590249	2647588502	2647589384		
Agathobacter rectale DSM 17629	650377936	Bacillota, Lachnospiraceae	GIT							
Aggregatibacter aphrophilus F0387	2537561617	Pseudomonadota, Pasteurellaceae	GIT			2537980074		2537979357		
Akkermansia muciniphila ATCC BAA-835	2619619234	Verrucomicrobiota, Akkermansiaceae	GIT							
Alistipes shahii WAL 8301	650377904	Bacteroidota, Rikenellaceae	GIT							
Anaerobaculum hydrogeniformans BAA-1850	2517487012	Synergistota, Acetomicrobiaceae	GIT							
Anaerobutyricum hallii DSM 3353	643886203	Bacillota, Lachnospiraceae	GIT							
Anaerococcus hydrogenalis DSM 7454	642979323	Bacillota, Peptoniphilaceae	GIT							
Anaerofustis stercorihominis DSM 17244	641736193	Bacillota, Eubacteriaceae	GIT							
Anaerostipes hadrus DSM 3319	2534681714	Bacillota, Lachnospiraceae	GIT							
Anaerotruncus colihominis DSM 17241	641736271	Bacillota, Oscillospiraceae	GIT							
Anaerovorax odorimutans DSM 5092	2524614531	Bacillota, Anaerovoracaceae	GIT							
Aneurinibacillus aneurinilyticus ATCC 12856	2563366762	Bacillota, Paenibacillaceae	GIT	2563974344			2647588858			

Organism Name	Genome ID	Taxonomy (Phylum/ family)	Isolation	NasA	NarG	NapA	NasB	NrfA	NirK	YtfE
Arcobacter butzleri JV22	649989908	Campylobacterota, Arcobacteraceae	GIT			650299545		650299534		
Bacteroides xylanisolvens XB1A	2938060881	Bacteroidota, Bacteroidaceae	GIT					2938062062		650523269
Barnesiella intestinihominis YIT 11860	2529292932	Bacteroidota, Barnesiellaceae	GIT							
Bifidobacterium longum infantis ATCC 15697	643348516	Actinomycetota, Bifidobacteriaceae	GIT							
Bifidobacterium adolescentis L2-32	640963015	Actinomycetota, Bifidobacteriaceae	GIT							
Bilophila wadsworthia 3_1_6	2562617176	Thermodesulfobacteriota, Desulfovibrionaceae	GIT							
Blautia hansenii DSM 20583	2773857866	Bacillota, Lachnospiraceae	GIT							
Brevundimonas diminuta 470-4	2531839707	Pseudomonadota, Caulobacteraceae	GIT							
Marvinbryantia formatexigens I-52	2562617090	Bacillota, Lachnospiraceae	GIT							
Butyricicoccus pullicaecorum 1.2	2537561731	Bacillota, Butyricicoccaceae	GIT							
Butyrivibrio crossotus DSM 2876	645951834	Bacillota, Lachnospiraceae	GIT							
Campylobacter coli JV20	648276627	Campylobacterota, Campylobacteraceae	GIT			648828586				
Capnocytophaga sp. oral taxon 335 F0486	2531839279	Bacteroidota, Flavobacteriaceae	GIT					2532916161		2532916476
Catenibacterium mitsuokai DSM 15897	643886110	Bacillota, Coprobacillaceae	GIT							
Cedecea davisae DSM 4568	2541046972	Pseudomonadota, Enterobacteriaceae	GIT		2541307490	643148105	2541309359			2541305324
Cetobacterium somerae ATCC BAA-474 (DSM 23941)	2558860278	Fusobacteriota, Fusobacteriaceae	GIT	2559417427		2559419040				
Christensenella minuta DSM22607	2836893620	Bacillota; Christensenellaceae	GIT							
Chryseobacterium taklimakanense H4753	2851049461	Bacteroidota, Weeksellaceae	GIT							
Citrobacter youngae ATCC 29220	2562617093	Pseudomonadota, Enterobacteriaceae	GIT							
Clostridioides difficile NAP07	647000227	Bacillota Peptostreptococcaceae	GIT							
Clostridium leptum DSM 753	641380427	Bacillota; Oscillospiraceae	GIT							

Organism name	Genome ID	Taxonomy (Phylum/ family)	Isolation	NasA	NarG	NapA	NasB	NrfA	NirK	YtfE
Collinsella intestinalis DSM 13280	2562617146	Actinomycetota, Coriobacteriaceae	GIT							
Coprobacillus cateniformis 29_1	649989924	Bacillota, Coprobacillaceae	GIT							
Coprococcus catus GD/7	650377925	Bacillota, Lachnospiraceae	GIT							
Corynebacterium ammoniagenes DSM 20306	647000230	Actinomycetota, Corynebacteriaceae	GIT		647491136					
Dermabacter sp. HFH0086	2541047015	Actinomycetota, Dermabacteraceae	GIT				2541450641			
Desulfitobacterium hafniense DCB-2 (DSM 10664)	2522572064	Bacillota, Desulfitobacteriaceae		643561201		643561201		643563553		2522799566
Desulfovibrio piger ATCC 29098 (DSM 749)	642979316	Thermodesulfobacteriota, Desulfovibrionaceae	GIT							
Dialister succinatiphilus	2513237349	Bacillota, Veillonellaceae	GIT							
Dorea formicigenerans ATCC 27755 (DSM 3992)	641736133	Bacillota, Lachnospiraceae	GIT							
Dysgonomonas mossii DSM 22836	651324027	Bacteroidota, Dysgonomonadaceae	GIT					651391164		
Edwardsiella tarda ATCC 23685	647000237	Pseudomonadota, Hafniaceae	GIT		647367413	647368492		647370560		647367540
Eggerthella lenta VPI 0255, DSM 2243	644736358	Actinomycetota, Eggerthellaceae	GIT			645024705				
Enterocloster clostridioformis WAL-7855	2648501893	Bacillota, Lachnospiraceae	GIT							
Enterococcus faecalis TX0104	643886065	Bacillota, Enterococcaceae	GIT	644294837						
Escherichia coli K-12, MG1655	2600254932	Pseudomonadota, Enterobacteriaceae	GIT	646314162	646313148	646314162	646315332	646316056		2648091051
Eubacterium siraeum DSM 15702	641736184	Bacillota, Oscillospiraceae	GIT							
Faecalibacterium prausnitzii A2-165	645951831	Bacillota, Oscillospiraceae	GIT							
Faecalitalea cylindroides ATCC 27803	2558860279	Bacillota, Erysipelotrichaceae	GIT							
Finegoldia magna ATCC 29328	641522627	Bacillota, Peptoniphilaceae	GIT							
Flavonifractor plautii ATCC 29863	2513237287	Bacillota, Oscillospiraceae	GIT							
Fusobacterium gonidiaformans ATCC 25563	645951804	Fusobacteriota, Fusobacteriaceae	GIT							

Organism Name	Genome ID	Taxonomy (Phylum/ family)	Isolation	NasA	NarG	NapA	NasB	NrfA	NirK	YtfE
Gordonibacter pamelaeae<br>7-10-1-b	650377943	Actinomycetota, Eggerthellaceae	GIT							
Haemophilus influenzae<br>NCTC8143	2660238341	Pseudomonadota, Pasteurellaceae	GIT			2661991786		2661992880		2661992282
Hafnia alvei<br>ATCC 51873 (all)	2513237377	Pseudomonadota, Hafniaceae	GIT		2514672033	2514672119	2514671483			
Helicobacter pylori<br>35A	2511231168	Campylobacterota, Helicobacteraceae	GIT							2514673188
Holdemania filiformis<br>DSM 12042	643886103	Bacillota, Erysipelotrichaceae	GIT							
Hominimerdicola aceti<br>(*Ruminococcus bicirculans*)	2585427614	Bacillota, Oscillospiraceae	GIT							
Klebsiella oxytoca<br>KA-2	2563367188	Pseudomonadota,<br>Enterobacteriaceae	GIT	2565779637	2565779642		2565779636			2533391056
Lacticaseibacillus rhamnosus<br>ATCC 21052	2534681855	Bacillota, Lactobacillaceae	GIT							
Limosilactobacillus reuteri<br>DSM 20016	640427118	Bacillota, Lactobacillaceae	GIT		2674094276					
Leuconostoc mesenteroides<br>ATCC 19254	643886109	Bacillota, Lactobacillaceae	GIT							
Listeria grayi<br>DSM 20601	2562617157	Bacillota, Listeriaceae	GIT							
Megamonas funiformis<br>YIT 11815	2513237399	Bacillota, Selenomonadaceae	GIT							
Mitsuokella multacida<br>DSM 20544	2562617158	Bacillota, Selenomonadaceae	GIT							
Mogibacterium timidum<br>ATCC 33093	2558860283	Bacillota, Anaerovoracaceae	GIT							
Morganella morganii<br>NLAE-zl-C84	2654588210	Pseudomonadota, Morganellaceae	GIT		2657241810		2657244419			2657241251
Neisseria macacae<br>ATCC 33926	651324075	Pseudomonadota, Neisseriaceae	GIT						651678257	651679500
Odoribacter laneus<br>YIT 12061	2513237280	Bacteroidota, Odoribacteraceae	GIT							
Oxalobacter formigenes<br>OXCC13	646206265	Pseudomonadota, Oxalobacteraceae	GIT							646260796
Paenibacillus rubiinfantis<br>MT18	2775507042	Bacillota, Paenibacillaceae	GIT	2776879590	2776877904		2776879044			651516875
Paenisporosarcina sp.<br>HGH0030	2541046999	Bacillota, Caryophanaceae	GIT							

Organism Name	Genome ID	Taxonomy (Phylum/ family)	Isolation	NasA	NarG	NapA	NasB	NrfA	NirK	YtfE
Parabacteroides johnsonii DSM 18315	642979358	Bacteroidota, Tannerellaceae	GIT							
Paraprevotella clara YIT 11840	2513237390	Bacteroidota, Prevotellaceae	GIT							
Parasutterella excrementihominis DSM 21040	651324084	Pseudomonadota, Sutterellaceae	GIT		651553556	651553087				
Parvimonas micra ATCC 33270	641380421	Bacillota, Peptoniphilaceae	GIT							
Pediococcus acidilactici DSM 20284	649989981	Bacillota, Lactobacillaceae	GIT							
Peptoniphilus asaccharolyticus UW 228, DSM 20463	2503982046	Bacillota, Peptoniphilaceae	GIT							2504128174
Phascolarctobacterium faecium DSM 14760	2756170237	Bacillota, Acidaminococcaceae	GIT							
Phocaeicola dorei DSM 17855	642979370	Bacteroidota, Bacteroidaceae	GIT							643123629
Phocaeicola vulgatus ATCC 8482	2886510283	Bacteroidota, Bacteroidaceae	GIT							640763088
Plesiomonas shigelloides NCTC 10364	2811995289	Pseudomonadota, Enterobacteriaceae	GIT			2813712859		2813713748		2813714213
Proteus mirabilis WGLW6	2537561878	Pseudomonadota, Morganellaceae	GIT		2539022274		2539021324			
Providencia alcalifaciens DSM 30120	642979317	Pseudomonadota, Morganellaceae	GIT			643148105		643148102		
Pseudomonas syringae DSM 1241	2523533564	from file NIH Human Microbiome Project_Master with genera	GIT	2523801047			2523800124			
Pyramidobacter piscolens W5455	647000302	Synergistota, Dethiosulfovibrionaceae	GIT							
Raoultella ornithinolytica 10-5246	2531839403	Pseudomonadota, Enterobacteriaceae	GIT	2533393522	2533393517		2533393523			
Roseburia intestinalis L1-82	2562617159	Bacillota, Lachnospiraceae	GIT							
Ruminococcus bromii L7660, ATCC 27255	2857962555	Bacillota, Oscillospiraceae	GIT							
Ruminococcus torques ATCC 27756	640963025	Bacillota, Lachnospiraceae	GIT							

Organism Name	Genome ID	Taxonomy (Phylum/ family)	Isolation	NasA	NarG	NapA	NasB	NrfA	NirK	YtfE
Segatella copri DSM 18205	2562617166	Bacteroidota, Prevotellaceae	GIT							
Selenomonas artemidis F0399	649989996	Bacillota, Selenomonadaceae	GIT		650189751			650190061		
Simonsiella muelleri ATCC 29453	2562617187	Pseudomonadota, Neisseriaceae	GIT		2563322591				2563322138	2563321330
Slackia exigua ATCC 700122 (DSM 15923)	645951837	Actinomycetota, Eggerthellaceae	GIT			646136908				
Staphylococcus warneri	645058825	Bacillota, Staphylococcaceae	GIT		645535819		645535822			
Streptococcus anginosus 1_2_62CV	649990001	Bacillota, Streptococcaceae	GIT							
Streptococcus infantarius ATCC BAA-102	641736171	Bacillota, Streptococcaceae	GIT							
Subdoligranulum variabile DSM 15176	2562617078	Bacillota, Oscillospiraceae	GIT							
Succinatimonas hippeii YIT 12066	649990019	Pseudomonadota, Succinivibrionaceae	GIT							
Sutterella megalosphaeroides 6FBBBH3	2833028006	Pseudomonadota, Sutterellaceae	GIT			2833028280				
Tannerella sp. 6_1_58FAA_CT1	2513237267	Bacteroidota, Tannerellaceae	GIT							
Thomasclavelia ramosa DSM 1402	641736198	Bacillota, Coprobacillaceae	GIT							
Turicibacter sp. HGF1	651324107	Bacillota, Turicibacteraceae	GIT	651509140						
Varibaculum cambriense DNF00696	2806310719	Actinomycetota, Actinomycetaceae	GIT		2807334903					
Veillonella atypica ATCC 17744 (DSM 20739)	2529292891	Bacillota; Veillonellaceae	GIT		2530405622					
Weissella paramesenteroides ATCC 33313	645058793	Bacillota, Lactobacillaceae	GIT							
Yokenella regensburgei ATCC 43003	2534682241	Pseudomonadota, Enterobacteriaceae	GIT	2537110358	2537113108					2537109616

a) Abbreviations: NasA = assimilatory nitrate reductase, NarG = respiratory nitrate reductase, NapA = periplasmic nitrate reductase, NasB = assimilatory nitrite reductase, NrfA=respiratory nitrite reductase. NirK = Copper-containing nitrite reductase. All nitrate reductases form nitrite from nitrate. NapA and NasB catalyze the conversion of nitrite to ammonia. NirK reduces nitrite to NO. NirS homologs were not found in any of the analyzed GIT bacteria. YtfE = Di-iron nitrite reductase. Amino acid sequences from biochemically well-characterized enzymes were used as model proteins for a BLASTp analysis in the IMG database (https://img.jgi.doe.gov/cgi-bin/mer/main.cgi). NasA: NAD(P)H dependent assimilatory NO_3^- reductase from *K. oxytoca* (Q06457); NarG: membrane-bound respiratory nitrate reductase from *E. coli* (BAA36094.1); NapA: periplasmic respiratory nitrate reductase from *E. coli* (BAA15989.2); NrfA periplasmic NO_2^- reductase from *E. coli* (P0ABK9); NirB: NADH- dependent assimilatory NO_2^- reductase from *E. coli* (NP_417824); NirS: NO forming NO_2^- reductase from *P. stutzeri* (P24040); NirK NO forming NO_2^- reductase from *P. chlororaphis* (Q06006); YtfE: Di-Iron NO forming NO_2^- reductase from *E. coli* (AAC77166.1)
